# Chaplaincy in a Free-Standing Psychiatric Hospital During the COVID-19 Pandemic

**DOI:** 10.1177/1542305021999981

**Published:** 2021-03-17

**Authors:** Angelika A. Zollfrank

**Affiliations:** Spirituality & Mental Health Program, McLean Hospital, Belmont, MA, USA

**Keywords:** Chaplaincy, COVID-19, mental health chaplaincy

## Abstract

This Special Issue discusses the results of the international COVID-19 survey that took place during the first wave of the pandemic. This contribution discusses chaplaincy in a psychiatric hospital during the COVID-19 pandemic. Chaplaincy vignettes with patients and interventions with staff are described, showing how chaplaincy changed and remained the same during this time. The focus here is on acknowledging disturbed and broken connections, as well as intervening to sustain community.

This Special Issue of the Journal of Pastoral Care and Counseling discusses the results of the international COVID-19 survey that took place during the first wave of the pandemic in late spring 2020. Usually when talking about chaplaincy in health care during the pandemic, the focus is on chaplaincy in general hospitals and residential care homes. This contribution discusses chaplaincy in a psychiatric hospital where Covid-19 affected patients and staff in ways, often unknown to the public.

Chaplaincy in psychiatry uniquely contributes to integrating spirituality and religion into mental health treatment. In one apt description psychiatrists’ goal with patients is “communication”, giving and receiving information. Mental health chaplains are focused on creating “communion”, a common space “where two people can authentically meet” (Elbaum, 2019). The lack of communion and the change to how human beings commune authentically have been key characteristics of the COVID-19 pandemic, leading to symptoms of anxiety disorders and depression having tripled throughout the world community (Czeisler et al., 2020). This paper reports how mental health chaplaincy in one hospital has adapted and continued to provide communion to people with severe mental illness (SMI).

Persons living with SMI already experience ex-communication, stigmatization, and disadvantage due to less physical health care, poverty, poor lifestyle choices, housing difficulties, lack of social connection (Druss, 2020), and “employment discrimination and criminalization” (CDC, 2021). During COVID-19 substance use disorders increased in persons with SMI by about 50% (Hamada & Fan, 2020). COVID-19 also has caused increased rates of “new onset of psychosis and exacerbation of symptoms in individuals with SMI” (Hamada & Fan, 2020) and social distancing caused stress leading to relapse of psychotic symptoms in 30% of persons with SMI (Muruganandam et al., 2020). Social deprivation is significantly associated with “depression, paranoid thinking, and suicide ideation” (Hamada & Fan, 2020) and moderately associated with mania, depression, paranoia and hallucinations via induction of stress” (Lazzari et al., 2020).

The following vignette describes a relapse of psychotic symptoms. A 45-year-old Protestant mother, with MDD and GAD, stated, “It’s all my fault. I gave it to them. My kids, my husband, my mother, they will all die from the virus. Everyone will die and I gave it to them. I feel so guilty. I destroyed my family. I cannot even talk with you.” In reality no one in her family was infected, but unmanageable fear of COVID had led to delusions. The chaplain asked if she thought it might help to pray together. “No, I don’t deserve prayer. I don’t deserve it and God knows it. It’s better for you to leave.” Any possibility for communion was destroyed by COVID. A week later, the patient tolerated the chaplain sitting with her and she accepted a prayer for grace and healing.

Often inpatient psychiatric units are places of safety offering community for those in acute crises. However, patients initially spent many days in Emergency Departments waiting for COVID test results and an available bed, making access to inpatient psychiatric treatment more burdensome. Delay in treatment combined with greater stressors and increased need for psychiatric care led to higher inpatient acuity. Slade (2020) reported a 131% increase in patient volume in an ambulatory psychiatric team. Psychiatric hospitals are not equipped to accommodate highly infectious patients. For patients lacking insight, on the other hand, social distancing has been a difficult norm. Hopelessness, depression, or willingness to self-harm or harm others in patients are also expressed in nonadherence to infection control protocols.

Once stabilized, the treatment team facilitates the transition back into the community. Due to COVID-19 day or weekend passes have become unavailable. This change has made discharges more anxiety-provoking, turbulent, less lasting, or all together impossible. Many community programs, group homes, and residential programs were closed for months, delaying patients’ discharge and recovery progression. Meanwhile support systems and communities have been irreversibly altered for many.

For example, a 73-year-old spiritual, not religious woman retired after a high-achieving professional life interrupted by severe bouts of depression and anxiety, had lost her brother and sister-in-law to COVID-19. The couple had been the patient’s familial, social, financial, and legal support system. “I have no one in the world anymore. It is terrifying. Why would I go on?” Normally the spiritual care plan would have aimed at reconnecting the patient with her Unitarian Universalist community. “I do better in person. Talking to you helps. I won’t do Zoom.” For a time, the chaplain and the patient together formed a connection that nurtured meaning-making.

SAMHSA reported that the US executive order to waive strict regulations regarding telehealth saved lives (SAMHSA, 2020). One advantage of partial and outpatient behavioral health services moving to Zoom has been that many more patients in communities with traditionally less access can get treatment. However, barriers exist for the elderly, less computer-literate, or less well-connected.

Persons with SMI are often isolated, yet also are searching for meaning and connection. Many psychiatric patients who depend on a weekly structure, felt the temporary closure of places of worship to be very difficult. While spiritual and religious communities can sometimes contribute to spiritual struggle and relational trauma, they are also a lifeline. These communities offer acceptance and belonging. They allow for participation that accommodates relational closeness as well as anonymity and distance. They offer validation beyond productivity, formal education, earning power, and competitiveness. They affirm the value, worth, and dignity of any person, if not interpersonally, then even more powerfully by a Higher Power, the Sacred, or God.

A 49-year-old woman carrying diagnosis of borderline personality disorder, dissociative disorder, depression, and chronic suicidality, said, “Sometimes I talk to my neighbor, but she is Born-Again and that just rubs me the wrong way. Having the Christmas service here on the unit was the highlight of my admission this time. I needed a worship service.” A Jewish man, who gathered with a group of patients outside during RoshHashana cried, “I never have not heard the Shofar on RoshHashana. I am so uplifted that even during COVID we can say the blessings and have the Shofar.”

In addition to inpatient groups, faith-specific services, and individual consultation, mental health chaplains have offered outpatient visits through Zoom. This new reality has also changed the boundaries between the role of the chaplain and the role of community spiritual and religious leaders. Mental health chaplains have also utilized smart phone apps and online resources to facilitate positive religious and spiritual coping more than prior to the pandemic. Former patients have reached out more frequently per email or phone.

Mental health chaplains connect patients with religious communities in many ways. For example, a 33-year-old female, Muslim Moroccan immigrant worked in a major hospital during the area’s COVID surge. Her husband was denied a Green card, shattering the couple’s hopes of reuniting. These stressors led to a relapse of anxiety and depression with paranoid ideation, panic attacks. A devout Muslim, the patient insisted on fasting. A prayer rug and Qur’an, connection through the language of faith, established trust. In response to the patient’s request, she and the chaplain also spent 15 minutes in prayer, each in her own tradition. The patient was able to share the details of her current situation. “You are like a sister. I have four sisters back home. We are sisters here, because we both have faith.” The spiritual care plan included assuring access to protein bars in the late evening, working with the medical team to determine a medication schedule that accommodated the daily fast, and frequent follow-up visits. Fasting during Ramadan connected this patient to the world-wide Muslim community as well as to her husband.

Without visitors already psychiatric patients’ often fragile social and familial relationships have been even more burdened. Family meetings continue via phone or on Zoom. Teams use hybrid rounding with one team member in the room and others on Zoom. For chaplaincy, this has led to less consistent presence at rounds, making email communication and charting even more important. On rare occasions large teams still gathered using the dining room as a meeting space.

For example, a 27-year-old Pentecostal man with schizophrenia, depression, and malignant catatonia was mute and – as he called it - “stiff”. He also exhibited excessive motor activity and echolalia. This life-threatening manifestation of catatonia receded slowly with treatment. Often, he was pacing or standing with a stare, while listening to gospel music. The chaplain regularly and unobtrusively made contact. Two months into the COVID crisis, his grandmother died. With the goal of mitigating the grief and helping the patient process, the chaplain invited the patient into the first longer conversation. The chaplain carefully watched the patient’s eyes and forehead above the mask. With patients recovering from catatonia, it is important to carefully observe the difference between the patient expressing himself versus the patient repeating the chaplain’s words in a perseverative loop. Did his grandmother live with him when he grew up? - “No.” Maybe he saw her in church? - “Yes, at church… every week… at church… my grandmother and my mother and I… we met at church… every week…” What a loss this must be. - “Tremendous! …It’s tremendous! … Tremendous …” And yes, he would like to listen to some old-fashioned gospel music together. As the music played, the patient came to life, “I remember this! I remember this from fourth grade! I remember this! In church. In fourth grade with grandmother! I remember this!” Attending the funeral was impossible. But acknowledging his grief in this way, eased the burden. Two months later, the same patient’s brother died of COVID. The patient acknowledged that this death could set him back. He reminisce about the life he shared with his brother and shared pictures. Processing grief is difficult for persons with SMI. Family, community, and ritual can help. Grieving in culturally and religiously established ways was impossible. Instead, the chaplain provided a brief memorial service with the patient’s team in attendance and his parents on Zoom.

Clinicians tend to focus on the psychiatric treatment and less on how present and past experiences of grief are at play. A 26-year-old devout Roman Catholic man was about to jump off the top of his apartment building, when his mother coaxed him to step back from the edge. The struggle with severe ADHD and depression was not new for him. “My father died when I was five years old. He died of a brain tumor.” Later the patient revealed his father’s anger and his abuse of his son. “And then he died.” The chaplain wondered what specific COVID stressors had triggered the suicide attempt? The young man shared his pride in his work in a large nursing home kitchen. “I had taught myself all the routines to get through my work day and get the job done. Not easy because of the ADHD. But I did it. I broke it down in small steps that I could repeat every day. But then everything changed. Not just one change but every few days a different protocol, a different way of doing things. I lost my routine and I was terrified … like if I didn’t sterilize something right, people would die because of me.” The thought that he could spread the deadly virus because of his ADHD was impossible to cope with. Long before he had received therapeutic and psychiatric help as a child, the rituals of the Roman Catholic Church and talks with the priest held this man’s mind together and gave him comfort. During COVID again, prayer and sharing his grief was helpful. Yes, people died of COVID-19 every day, but not through his actions. Just as much as his father had died of the brain tumor, not through anything he did or didn’t do. God knew this. “I knew I had to talk to the chaplain. With time and prayer, I will figure it out again.”

Surprisingly rare so far has been chaplaincy care for patients who were also healthcare providers. One case stands out, however. A nursing aid, who had worked in an ICU and care for COVID-19 patients, had brought the virus home. She and her husband recovered relatively quickly. Her elderly mother-in-law died from COVID. The accumulative burden of grief of three or four patients daily dying in the ICU and her guilt relative to her mother-in-law’s death, became too much for this woman. After becoming severely depressed and suicidal, she was hospitalized. The chaplaincy intervention focused on helping this woman, who was also training to become a nurse with goal of working in palliative care, to accept that cure is not always possible. Over the course of several visits, she was able to affirm her sense of calling to palliative care, and she was able to begin to differentiate her self-worth and contribution as a healthcare provider from the outcomes that were beyond her control. Her family’s support and her conversations with a forgiving and compassionate God became key in her recovery.

Overall, during COVID-19 patients and staff expressed an increased need for spiritual care. Facilities staff stopped the chaplain to lament the fact that the hospital had no designated prayer space. The chaplain prayed in hallways and staircases with staff who had lost a family member, had sick loved ones, or spouses who had lost their jobs. Many caregivers were worried about transmitting the virus, with COVID-19 patients in their patient care area. “I can’t tell them at home that I work with COVID patients. They think I’m ok because this is a psychiatric hospital.” Chaplaincy, embedded and well known already by hospital staff, responded to the particular kind of distress during each week of the unfolding crisis. Chaplaincy appreciated the stress caused by adapting to a changed reality, acknowledged shock and uncertainty, inspired gratitude, and offered support when grief or frustration sat in. Spiritual care interventions were designed to allow space for staff to express exhaustion, strengthen each other, and celebrate successful adaptation. Staff support became a greater focus while patient care continued at similar and increased levels.

Tangibles sings of care became important. For example, handing out prayers and daily reflection booklets, particularly to those staff known to be Christian and unable to attend their churches was much appreciated. In daily staff rounds, the chaplain asked Haitian and Latinx cleaning and nutrition staff regularly about their health, their family’s health and employment situation. When the stress of the COVID-19 pandemic was paired with the acknowledgment of the pandemic of racial injustice, chaplaincy prioritized checking in with black and brown staff and patients. All employees were offered special caregiver blessing cards in Creole, Spanish, and English. Caregiver blessings were offered in previous years, but during the pandemic, sharing one’s pain became more important: a long-awaited baby died only with parents present, rendering the extended family helpless and bereft. An elderly parent’s care could not be assured in the same way. A working mother juggling children’s education and a professional life while loved ones were lost without funerals. And many shared the joys and sorrows of celebrating the holidays without family gatherings.

Staff support also became a source of constant creativity. For example, on April 30, National Poem in Your Pocket Day in the U.S., the chaplain selected, printed, and rolled up poems about loss, grief, spring-time, new life, hope, and wisdom in times of adversity. In one day, the chaplain made contact with 260 staff throughout the institution. Each person was invited to pick a poem from a large bowl. If the poem someone picked, did not resonate, they were encouraged to trade with another person. Smiles and tears, sharing poetry and putting poems in one’s pocket became the highlight of the day. In the same manner, the chaplain distributed finger labyrinths, Christmas ornaments, and Hannukah dreidels. During Spiritual Care Month, staff and patients participated in an event “Planting Hope”, during which 250 daffodil bulbs were hidden on the grounds of the hospital. The chaplain also co-led peer support groups focused on spiritual care on Zoom for those working remotely. In-person staff support groups on the units continued per request. It became obvious that spiritual care offered a unique voice and language, which resonated with staff and leadership alike. The chaplain was asked to offer a weekly 5-8 minute-video message to all staff. Since July 2020 each of these weekly “SPIRIT MATTERS” video messages received 2000 hits on the hospital intranet highlighting a significant, previously unmet need among staff for meaning-making and reflection. Overall, chaplaincy developed from the model of embedded, unit-based spiritual care support for staff to institution-wide interventions, and effective virtual interventions that reaches patients and staff on site and remotely. Throughout the pandemic, mental health chaplaincy has provided acknowledgment of disturbed or broken connections. It has renewed community based on shared values, beliefs, faith. And the web of spiritual care connections built during COVID-19, has offered grounding, sustenance, and meaning in a troubled time.

## References

[bibr1a-1542305021999981] Centers for Disease Control and prevention. (2021, January). *Mental Health: Household Pulse Survey*. https://www.cdc.gov/nchs/covid19/pulse/mental-health.htm

[bibr1-1542305021999981] Czeisler M. E.Lane, R. I., Petrosky, E., Wiley, J. F., Christensen, A., Njai, R., Weaver, M. D., Robbins, R., Facer-Childs, E. R., Barger, L. K., Czeisler, C. A., Howard, M. E., & Rajaratnam, S. M. W. (2020). Mental health, substance use, and suicidal ideation during the Covid-19 pandemic – United States. Morbidity and Mortality Weekly Report, 69(32), 1049–1057. https://www.cdc.gov/mmwr/volumes/69/wr/mm6932a1.htm 32790653 10.15585/mmwr.mm6932a1 PMC7440121

[bibr2-1542305021999981] Druss B. G. (2020). Addressing the COVID-19 pandemic in populations with serious mental illness. JAMA Psychiatry. Advance online publication. 10.1001/jamapsychiatry.2020.0894 32242888

[bibr3-1542305021999981] Elbaum A. (2019). In the lions' den: How chaplaincy can inform psychiatry. Academic Psychiatry, 43, 645–647. 31482395 10.1007/s40596-019-01109-8

[bibr4-1542305021999981] Hamada K.& Fan, X. (2020). The impact of COVID-19 on individuals living with serious mental illness. Schizophrenia Research, 222, 3–5. 10.1016/j.schres.2020.05.054 32473931 PMC7250778

[bibr5-1542305021999981] Lazzari C.Shoka, A., Nusair, A., & Rabottini, M. (2020). Psychiatry in time of Covid-19 pandemic. Psychiatria Danubina, 32, 229–235. 10.24869/psyd.2020.229 32796791

[bibr6-1542305021999981] Muruganandam P.Neelamegam, S., Menon, V., Alexander, J., & Chaturvedi, S. K. (2020). COVID-19 and severe mental illness: Impact on patients and its relation with their awareness about COVID-19. Psychiatry Research, 291, 113265. 10.1016/j.psychres.2020.113265 32763536 PMC7322460

[bibr7-1542305021999981] SAMHSA. (2020, December). *Executive order saving lives through increased support for mental and behavioral health needs report*. https://www.samhsa.gov/sites/default/files/saving-lives-mental-behavioral-health-needs.pdf

[bibr8-1542305021999981] Slade R. (2020). *Inside Boston’s looming mental health crisis*. https://www.bostonmagazine.com/health/2020/090/22/mental-health-boston/print

[bibr9-1542305021999981] World Health Organization and Calouste Gulbenkian Foundation. (2014). *Social determinants of mental health (Geneva and Lisbon: 2014)*. https://apps.who.int/iris/bitstream/handle/10665/112828/9789241506809_eng.pdf;jsessionid=17FC3EFB86970FAA832FF84342E895D9?sequence=1

